# SMTdb: A Comprehensive Spatial Meta-Transcriptome Resource in Cancer

**DOI:** 10.1093/molbev/msaf263

**Published:** 2025-10-15

**Authors:** Weiwei Zhou, Qingyi Yang, Jiyu Guo, Si Li, Minghai Su, Feng Leng, Tingyu Rong, Jingyi Shi, Yueying Gao, Tiantongfei Jiang, Juan Xu, Yongsheng Li

**Affiliations:** College of Bioinformatics Science and Technology, Harbin Medical University, Harbin 150081, China; School of Interdisciplinary Medicine and Engineering, Harbin Medical University, Harbin 150081, China; School of Interdisciplinary Medicine and Engineering, Harbin Medical University, Harbin 150081, China; School of Interdisciplinary Medicine and Engineering, Harbin Medical University, Harbin 150081, China; School of Interdisciplinary Medicine and Engineering, Harbin Medical University, Harbin 150081, China; College of Bioinformatics Science and Technology, Harbin Medical University, Harbin 150081, China; College of Bioinformatics Science and Technology, Harbin Medical University, Harbin 150081, China; School of Interdisciplinary Medicine and Engineering, Harbin Medical University, Harbin 150081, China; College of Bioinformatics Science and Technology, Harbin Medical University, Harbin 150081, China; School of Interdisciplinary Medicine and Engineering, Harbin Medical University, Harbin 150081, China; College of Bioinformatics Science and Technology, Harbin Medical University, Harbin 150081, China; College of Bioinformatics Science and Technology, Harbin Medical University, Harbin 150081, China; State Key Laboratory of Frigid Zone Cardiovascular Diseases (SKLFZCD), Harbin Medical University, Harbin, Heilongjiang 150081, China; School of Interdisciplinary Medicine and Engineering, Harbin Medical University, Harbin 150081, China; State Key Laboratory of Frigid Zone Cardiovascular Diseases (SKLFZCD), Harbin Medical University, Harbin, Heilongjiang 150081, China

**Keywords:** spatially resolved transcriptomics, single-cell omics, database, tumor microenvironment, microbiota, immune cells

## Abstract

Microorganisms have been detected in various tumors, and research on the tumor microbiome has received increasing attention. However, the investigation of the cancer microbiome at the spatial resolution level remains a challenging issue. The emergence of spatially resolved transcriptomics technology has enabled to map transcripts at the single-cell resolution in various cancer types. Here, we constructed a comprehensive spatial meta-transcriptome resource by manually curating 203 fresh frozen slices from 20 cancer types encompassing 334,253 spots and 1,908,646 cells. A spatial meta-transcriptome database (SMTdb; http://bio-bigdata.hrbmu.edu.cn/SMTdb/) was constructed to provide detailed insights into the abundance, distribution, and enriched tumor microenvironment (TME) regions of 1,218 microbiota in spatial tissue slices. SMTdb enables to explore the vast interactive data of spatial distribution and expression of microbiota, provides host gene modules associated with certain microbiota, and contains data on the co-occurrence between the microbiota and immune cells within the TME. The atlas resource serves as a comprehensive and structured platform to investigate the interactions between microbial ecosystems and hosts in cancer.

## Graphical Abstract

**Graphical Abstract:**
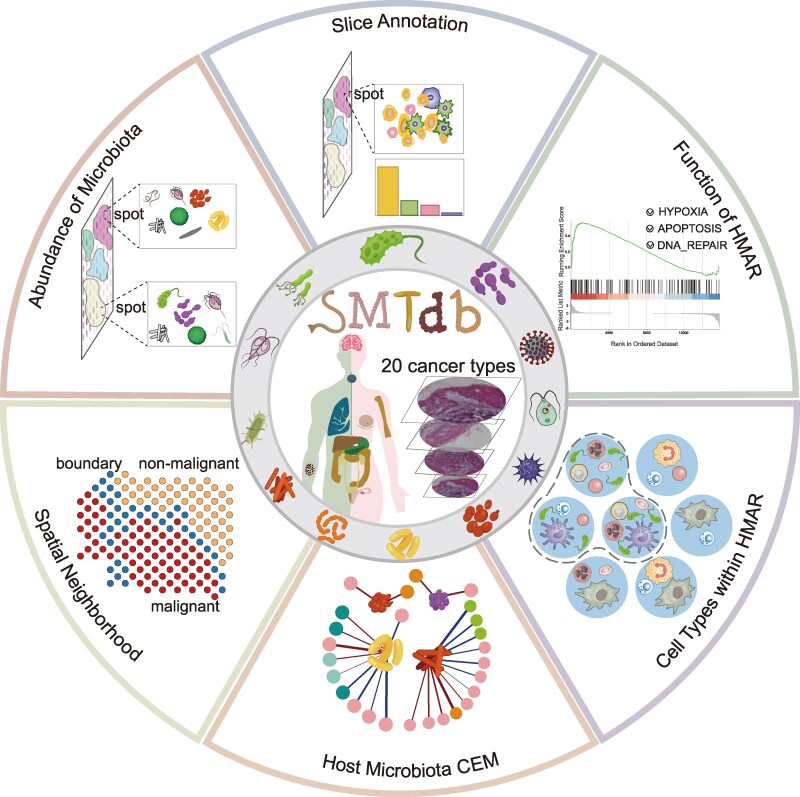

## Introduction

The tumor microenvironment (TME) is complex milieu surrounding a tumor and comprises multiple components, including tumor cells, stromal tissues, and the extracellular matrix. Diverse factors secreted by immune and nonimmune cells in the TME drive the inflammatory, immunosuppressive, and proangiogenic internal environments of tumors (Jin and Jin 2020). Numerous studies have confirmed that the interactions between tumor cells and immune cells play a critical role in the TME (Mao et al. 2021; Ma et al. 2023). Additionally, emerging evidence shows that microorganisms are present in various tumors and can influence cancer progression (LaCourse et al. 2021), metastasis (Bullman et al. 2017; Parhi et al. 2020; Fu et al. 2022), immune surveillance (Jin et al. 2019; Riquelme et al. 2019), and drug resistance of tumors (Geller et al. 2017; Yu et al. 2017). Hence, understanding the composition of the microbiota and its interaction with the tumor cells in the TME is vital to elucidate the molecular mechanisms underlying tumor progression.

The development of single-cell RNA sequencing (scRNA-seq) technology has enabled to examine the relationships between the microbiota and tumor cells in the TME. A recent study characterized the features of the intratumoral microbiota and revealed the most abundant bacterial orders in intrahepatic cholangiocarcinoma (Chai et al. 2023). Spatial transcriptomics (ST) technology allows to determine the spatial distribution and co-occurrence of different cell types and microbiota in tissue slices. For example, by using this technology, host–microorganism interactions have been observed in oral squamous cell carcinoma and colorectal cancer (CRC; Galeano Niño et al. 2022). Meta-transcriptomics analysis, which captures the global RNA expression of hosts and diverse microbial taxa (bacteria, archaea, fungi, and viruses), is being increasingly used to elucidate host–microbiota interactions and provide comprehensive insights into the functional states under specific host contexts (Nejman et al. 2020; Becattini et al. 2021; Dohlman et al. 2022; de Celis et al. 2024; Wu et al. 2024). However, attempts are just emerging, and to date, no study has explored the spatial distribution pattern of the microbiota and their interactions with immune cells in cancer.

In this context, we constructed a comprehensive spatial meta-transcriptome resource, designated spatial meta-transcriptome database (SMTdb), by integrating scRNA-seq and ST data. We manually curated 203 fresh frozen (FF) slices from 20 cancer types encompassing 334,253 spots. To assess the cell-type composition of spots, more than 1,900,000 cells from paired scRNA-seq and additional high-quality, cancer-matched scRNA-seq datasets were collected as reference. SMTdb provides significant information regarding the abundance, distribution, and enriched TME regions of 1,218 microbiota in spatial tissue slices. It also offers multiple analytical modules that allow users to interactively investigate the spatial distribution and expression of microbiota, host gene modules associated with certain microbiota, and data regarding the co-occurrence between the microbiota and immune cells within TME. As the first spatial microbiota-TME analysis and data resource, SMTdb can promote studies on understanding the roles of microorganisms in tumor development and progression and the related host immune response.

## Materials and Methods

### Data Collection

We collected raw FF ST data from the Gene Expression Omnibus database (Barrett et al. 2012) and public studies, including 120 tumor tissue slices and 83 peritumoral tissue slices from 20 cancer types (Table S1). A total of 1,908,646 cells from paired scRNA-seq and additional high-quality, cancer-matched scRNA-seq datasets (Zhou et al. 2024a) were matched to each slice as the reference data to determine the cell-type composition of spots.

### Microbiota Abundance in Spatial Slices

The STM, a genome sequence-based pipeline, was used to extract microbiota (Table S2) from ST datasets (Lyu et al. 2023). Briefly, reads that did not map to the host genome were first filtered and denoised, followed by BLAST alignment to the NCBI Nucleotide database (https://www.ncbi.nlm.nih.gov/nucleotide/); this process generated spatially resolved microbial abundance matrices and host gene expression profiles. Next, each spot was annotated with specific taxonomic labels if the unique molecular identifier (UMI) was greater than 0. The subsequent analyses of the microbiota were based on the abundance matrix.

### Processing and Clustering of ST and scRNA-Seq Data

To obtain the spatial transcriptome expression of each spot, the raw fastq files were processed with the SpaceRanger tool (version 2.0, 10 × Genomics) and mapped to the human reference genome (GRCh38). The Seurat package in R software was used for subsequent analyses (Butler et al. 2018). This analysis retained only those spots that coincided with tissue slices. The retained spots were further filtered by retaining those with at least 200 detected genes and excluding those with excessively low or high gene counts or with mitochondrial gene content exceeding 10% (Li et al. 2025). Genes with fewer than 10 read counts or expressed in fewer than 2 spots were also excluded. To normalize the raw counts, we adopted the strategy of regularized negative binomial regression, specifically the SC transform (Hafemeister and Satija 2019). Principal component analysis (PCA) was then applied to achieve dimensionality reduction. The Louvain algorithm (resolution = 0.8) was used to generate transcriptome clusters. To identify the marker genes associated with each cluster, the “findAllMarkers” algorithm was executed with the following parameters: “min.pct = 0.25, logfc.threshold = 0.25.” To identify spatially variable genes (SVGs), the function “FindSpatiallyVariableFeatures” in the Seurat package was used to measure the complex expression patterns of genes (SVGs; false discovery rate [FDR] < 0.05 and I > 0).

To analyze the scRNA-seq datasets, a uniform analytical pipeline was adopted using the Seurat package. Cells with high mitochondrial gene expression or abnormal UMI counts were excluded, and only those cells with 200 to 10,000 detected genes, UMI count >500, mitochondrial gene content <10%, and hemoglobin gene content <10% were retained. Genes with expression greater than 1% of total reads in more than 10% of capture locations were considered outliers and removed. Subsequently, the raw UMI counts were normalized using the SC transform method. Following this preprocessing step, highly variable genes were identified to facilitate the distinction of cellular heterogeneity. PCA was then performed to enable robust clustering of the cells.

### Cell-Type Annotation for the scRNA-Seq Data

A total of 363 canonical marker genes were manually recorded for 42 cell types from our previous study (Table S3; Jiang et al. 2023). The markers were used as the reference atlas for ScType, a fully automated and ultra-fast cell–type identification method, to assign cell types to each cell (Ianevski et al. 2022). InferCNV (version 1.2.1; Patel et al. 2014) was employed to identify malignant cells based on copy number variations (CNVs), with immune cells used as reference cells.

### Cell-Type Decomposition Analysis of the ST Data

In STMdb, robust cell-type decomposition (RCTD) was used to map the cell types found in the reference scRNA-seq dataset to the ST data. The standard RCTD analysis pipeline was followed for the reference and Visium ST data in the full doublet mode. Based on the deconvolution results, we assigned each spot to the cell type with the highest predicted proportion.

### Identification of Differentially Expressed Genes (DEGs)

To analyze how bacterial presence influences gene expression in tumors, we divided the malignant, boundary, and stromal spots into two groups according to bacterial presence in the spots and then conducted differential gene expression analysis. The Wilcoxon rank-sum test was used to assess the statistical significance of DEGs, and the FDR method was applied for correct the *P*-values. Genes with an FDR value of <0.05 and a fold change (FC) value of >1.5 were considered significantly upregulated.

### Spatial Colocalization of Cell Types and Microbiota

To assess the spatial colocalization between host cells and microbiota, RCTD was applied to infer cell-type composition across ST spots based on a reference scRNA-seq dataset. For bacteria-enriched spots, the cell-type composition was analyzed to identify host cell populations potentially involved in spatial colocalization with the microbiota.

### Identification of Spatial Neighborhood

To elucidate the complex spatial TME, we utilized Cottrazm (Xun et al. 2023) to map the microenvironment at the tumor boundary. The SME normalization algorithm from the stLearn package (Pham et al. 2023) was used to adjust gene expression based on the spot image matrix, resulting in a morphologically adjusted gene expression matrix (Morph), and spatial spots were clustered through the KNN algorithm from the Seurat package. Immune-related gene signatures were scored in the Morph matrix to define a normal tissue expression score (NormalScore) for each spot. Cottrazm selects a reference based on the highest median NormalScore within the cluster. InferCNV (Patel et al. 2014) was utilized to assess CNV levels for the remaining spots.

Hierarchical clustering was performed to categorize spatial spots into clusters to differentiate malignant spots from stromal spots. The CNV scores of each spot were incorporated into the Seurat object, and spots with high median CNV scores were initially defined as core malignant spots. Cottrazm calculated centroids for malignant and normal clusters and determined the proximity of each spot to these centroids. Spots were labeled as malignant or boundary based on their relative distances. The method arranges spatial spots on hexagonal lattices and defines neighboring spots by using Manhattan distances.

### Identification of Spatial Coexpression Modules

To identify spatial coexpression network modules, hdWGCNA (Morabito et al. 2023) was used to analyze the ST data. The MetaspotsByGroups() function was used to establish metaspots separately for each Seurat cluster, and the SetDatExpr() function was applied to construct the metaspot expression matrix. The soft power was tested by the TestSoftPowers() function, and the optimal threshold was determined. The ConstructNetwork() function was utilized to construct gene coexpression network modules.

### Functional Analysis

To predict the function of the microbiota-enriched regions, we identified DEGs using the Wilcoxon rank-sum test (FDR < 0.05 and FC > 1.5) separately for microbiota-enriched and nonmicrobiota-enriched regions. To assess the functions of bacteria present in the enriched regions, we estimated single-sample gene set enrichment analysis scores (Hanzelmann et al. 2013) for immune-related signatures (Li et al. 2020) and cancer hallmarks (Liberzon et al. 2015; Table S4). Additionally, a hypergeometric test was performed to identify the functions significantly upregulated in the microbiota-enriched regions.

### Spatial Correlation Between Host Genes and the Microbiota

Microbiota-gene coexpression modules in slices were assessed based on their expression at each spatial spot. For any pair of bacteria *i* and gene *j*, we first obtained the probabilities $E_{i}=[ei_{1},ei_{2},\ldots ,ei_{n}]$ and $E_{j}=[ej_{1},ej_{2},\ldots ,ej_{n}]$ that were observed across *n* spots, which represented the expression of bacteria and genes in those spots. Next, Spearman's correlation coefficient *R* was calculated according to the following formula:


$$R=\frac{\sum_{k=1}^{n}\;(E_{ik}-\bar{E_{i}})(E_{\;jk}-\bar{E_{j}})}{\sqrt{\sum_{k=1}^{n}\;{\left(E_{ik}-\bar{E_{i}}\right)}^{2}}\sqrt{\sum_{k=1}^{n}\;{\left(E_{\;jk}-\bar{E_{j}}\right)}^{2}}}$$

where $\bar{E_{i}}=\frac{1}{n}\sum_{k=1}^{n}\;E_{ik}$ and $\bar{E_{j}}=\frac{1}{n}\sum_{k=1}^{n}\;E_{\;jk}$ and *n* is the number of spots in the given slice.

For each bacterial taxon, all positively correlated genes (*R* > 0.1) were considered coexpression modules. We also investigated the functions influenced by the module, by using the hypergeometric test for Gene Ontology-biological process.

### Survival Analysis

To evaluate the association between microbial abundance and clinical prognosis of cancer patients, we collected clinical data from The Cancer Genome Atlas (TCGA; Tomczak et al. 2015), and the microbial abundance profile of TCGA samples was obtained from BIC database (Chen et al. 2023a). Next, we conducted univariate and multivariate Cox regression analyses. Additionally, patients were stratified into high- and low-abundance groups according to the median abundance of microorganisms in each cancer type, and survival differences between the two groups were assessed using the Kaplan–Meier method.

## Results

### Spatial and Single-Cell Transcriptomes Across Cancer Types

**Fig. 1.**
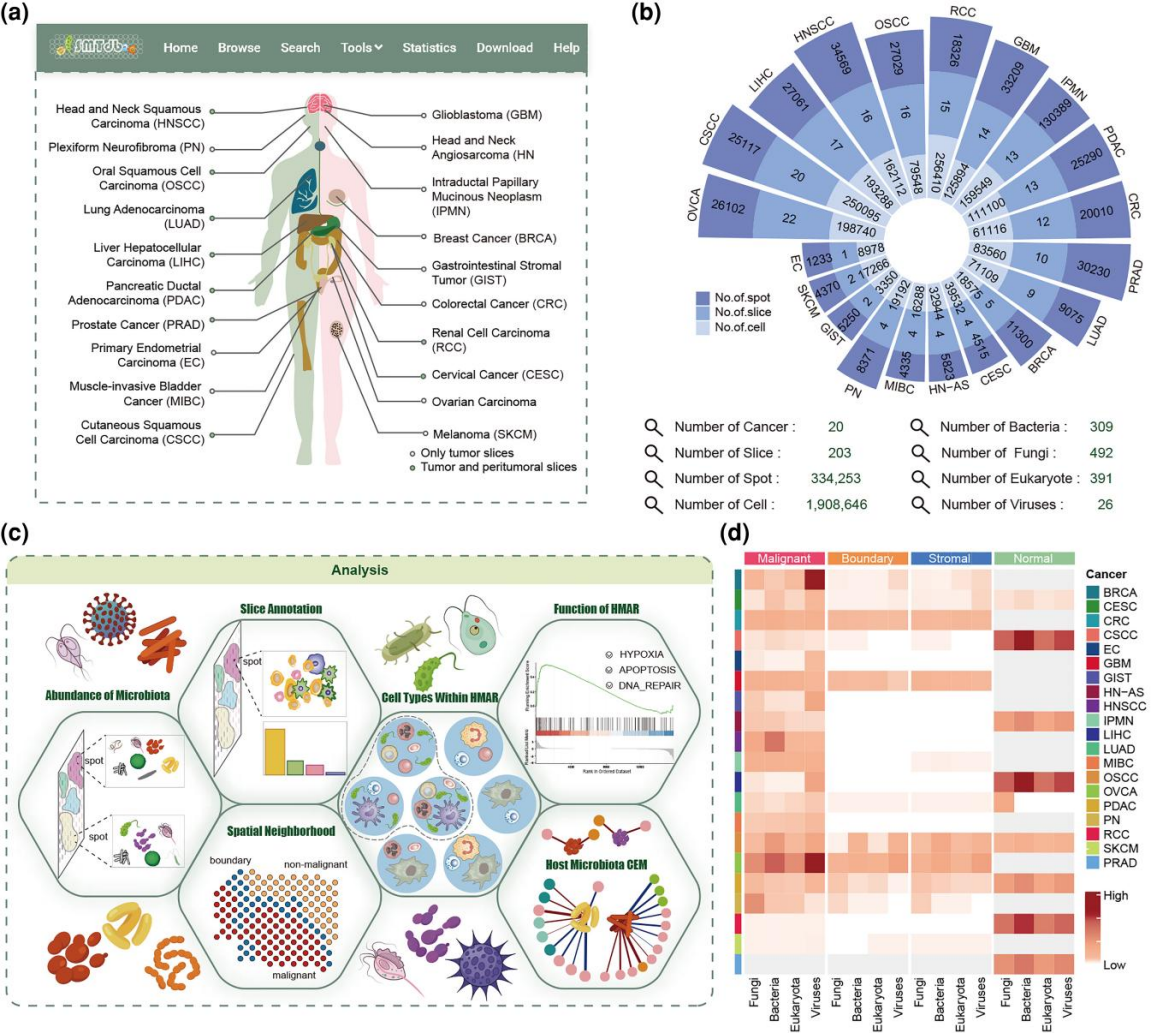
Overview for SMTdb construction. a) and b) Multiomics data collection. c) Functional analysis modules in SMTdb. (d) Microbiota abundance across cancer types.

To construct a comprehensive spatial meta-transcriptome resource for cancer, we manually collected the data of FF tissue samples based on a literature search over the past 5 years (Fig. 1a). FF samples are considered the gold standard for spatial genomics research, as they provide higher-quality DNA and RNA data compared to formalin-fixed and paraffin-embedded samples. Following strict filtering and uniform processing, we assembled a dataset comprising 203 tissue slices from 20 cancer types, including 120 tumor slices and 83 normal slices, with a total of 334,253 spots. The tumor slices and the normal slices contained an average of 1,800 and 1,600 spots per sample, respectively (Fig. 1b, Table S1).

To analyze the composition of the immune microenvironment in spatial tissue slices, we collected paired and additional high-quality scRNA-seq data for cell-type annotation by using marker genes. “Paired” implies data derived from the same tissue sample as that used for ST, while “high-quality” indicates high-quality, cancer-matched datasets curated from our previous study (Zhou et al. 2024; Table S5). After rigorous quality control and filtering, we constructed a pan-cancer single–cell atlas containing 1,908,646 cells across 42 cell types, including 187,114 tumor cells, 1,077,641 immune cells, and 644,071 stromal cells (Fig. 1b, Table S1). Finally, by using the corresponding single-cell atlas for each cancer type, we assigned cell types to each spot in tissue slices.

### Spatial Meta-Transcriptome for Cancer

By using the constructed ST atlas, we applied the analysis pipeline SMT (a method for extracting microbial sequences from ST data and assigning taxonomic labels) to assess microbial abundance in each spot (Fig. 1c, Fig. S1). The microbiota in the tissue slices were predominantly bacteria, fungi, and eukaryotes, along with a small amount of viruses (Fig. 1d, Fig. S2a).

A total of 26 viruses, 309 bacterial, 391 eukaryotic, and 492 fungal species were identified (Fig. S2b). Notably, significant differences were observed in the microbiota enriched in each cancer. *Citrobacter* was enriched in CRC, glioblastoma, muscle-invasive bladder cancer, renal cell carcinoma, and ovarian carcinoma, *Acidovorax* was predominantly enriched in breast cancer, and *Helicobacter* was enriched in gastrointestinal stromal tumor, suggesting microbial diversity and specificity across different cancer types (Fig. S2a and d). Compared to normal tissues, tumor tissues also exhibited a greater enrichment of the microbiota implicated in cancer development and progression (Jiang et al. 2015; Stasiewicz and Karpiński 2022; Zong et al. 2023), including *Candida*, *Agaricus*, and *Malassezia* (Fig. S2c). Our analysis constructed a comprehensive atlas of microbial distribution and abundance in diverse spatial contexts, providing a foundation for investigating the interactions between the microbiota and TME.

### User Interface Overview

We present SMTdb, a comprehensive data portal offering the exploration and visualization of microbial spatial distributions and their interactions with the TME. To enhance usability, SMTdb provides versatile functional panels, supporting overview and data exploration.

**Fig. 2.**
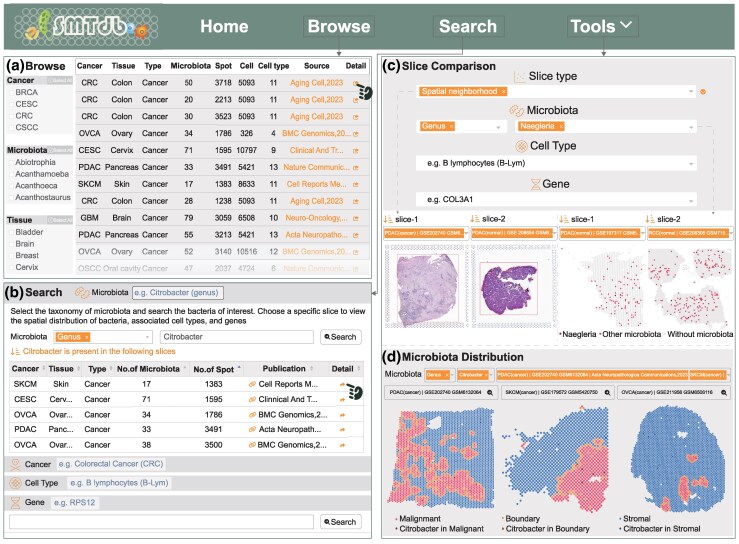
User interface of the SMTdb database. a) The “Browse” page allows users to filter slices of interest using the buttons located on the left sidebar. b) Example results generated using “Citrobacter” as a key term on the “Search” page. c) On the “Slice Comparison” page, users can analyze the similarities and differences across multiple features between any two slices. d) On the “Microbiota Distribution” page, users can visualize the spatial distribution of specific microbiota within different neighborhood regions across multiple slices.

The “Browse” page offers a slice list displaying cancer types, number of microbiota, spots, and cell types. Users can filter the table according to cancer type, tissue, or microbiota of interest and select a slice to access more information by pressing the “Detail” button (Fig. 2a).

The “Search” page has four search modes, allowing users to query specific microbiota, cancers, cell types, or genes. SMTdb filters slices to deliver tailored results. For example, Fig. 2b highlights slices containing “Citrobacter.”

The “Tools” page provides two interactive functions: “Slice comparison” and “Microbiota distribution.” The former displays variations in the distribution of characteristics such as spatial neighborhoods, microbial distribution, cell-type composition, and gene expression between two slices. Users can select any two spatial slices to understand the associations and differences between any features of interest within the slices (Fig. 2c). “Microbiota distribution” is designed for rapid comparison of a selected microbiota across multiple spatial slices. Users can view the spatial distribution of the specific microbiota in slices. Moreover, quick links are provided for users to access more details related to the microbiota in the selected slice (Fig. 2d).

### Slice Annotation

**Fig. 3.**
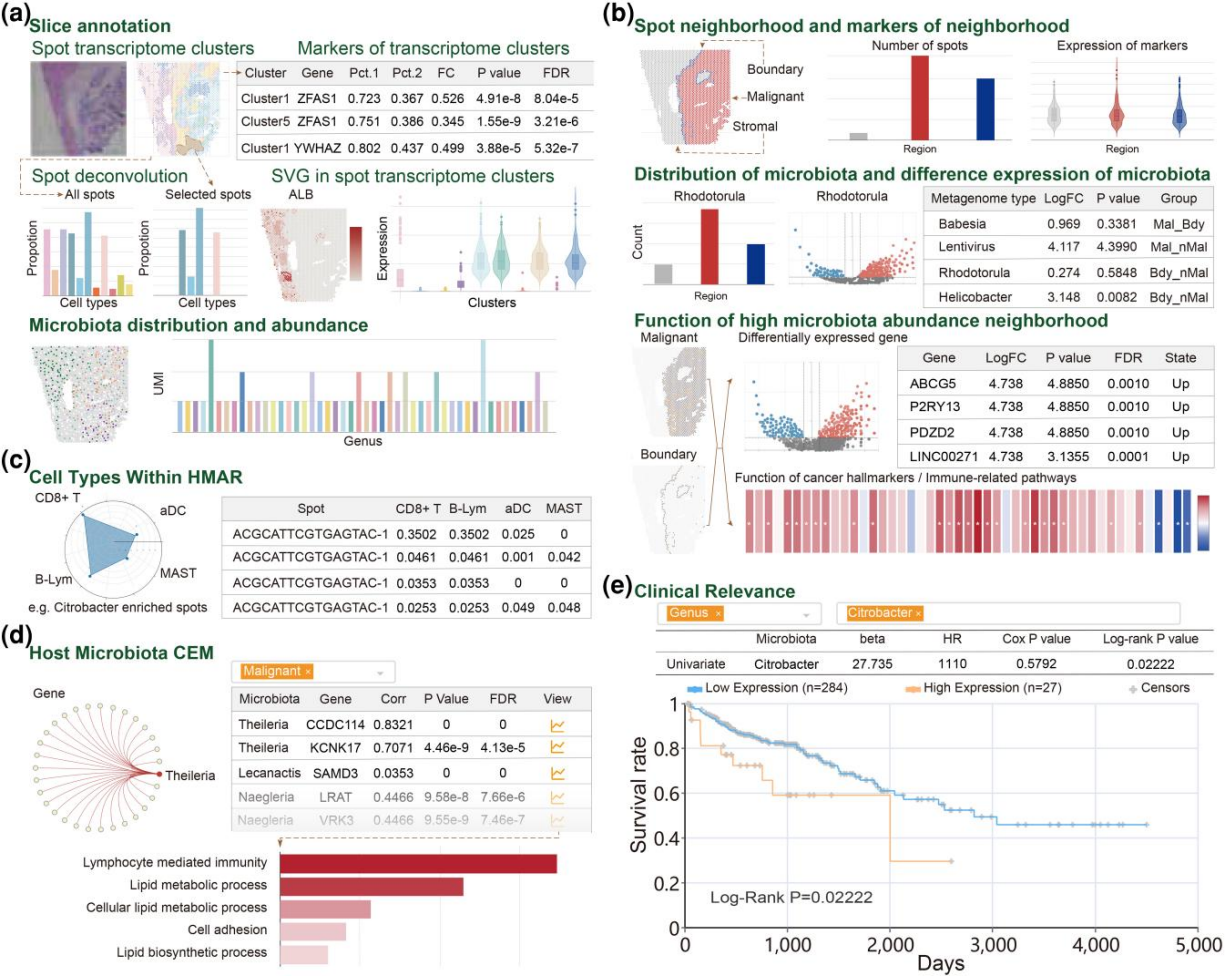
Analytical modules in SMTdb. a) Slice annotation module offers transcriptomic clusters, marker information of clusters, deconvolution of spots, and the microbiota abundance in tissue slices. b) Spatial neighborhood module clarifies the distribution of spots in the spatial slice and identifies differentially regulated genes and microbiota-enriched functions in malignant and boundary regions (cancer hallmarks for malignant spots and immune pathways for boundary spots). c) Co-occurrence module shows immune cell types that co-occur with bacteria in the TME across the spatial context. d) Gene modules regulated by bacteria in the host and the biological functions regulated by these modules in different regions. e) Kaplan–Meier plot from CRC patients of the TCGA cohort based on *Citrobacter* abundance, showing the classification of patients into the high- and low-expression groups based on the median value for analysis.

SMTdb annotated multiomics data for each tissue slice. First, SMTdb identified the marker genes of the transcriptome clusters. Users can browse the expression of genes at specific spatial locations. SMTdb also provided SVG for each slice, facilitating an understanding of the cellular states and functions unique to particular spatial region (Fig. 3a). Additionally, based on the scRNA-seq data, SMTdb provides the composition of cell types for each spot, enabling users to select specific spot or region for determining cell localization in tissue slices (Fig. 3a). Finally, the data related to the microbiota within spatial slices were also integrated. Users can explore the spatial distribution and abundance of microbiota of interest across an individual spot (Fig. 3a).

### Functions of Regions With High Bacterial Abundance

SMTdb defines malignant and boundary regions for each tissue slice. The malignant regions contain cells with the most prominent oncogenic features, whereas the boundary regions include the outermost layer of malignant cells in a solid tumor and spatially adjacent stromal cells; the boundary regions are frequently infiltrated by immune cells (Fig. 3b). SMTdb exclusively explores the cancer hallmarks regulated by microbiota-enriched spots in the malignant regions and characterizes the activity of immune pathways in the boundary regions. Users can easily access these results through various visualization features, such as volcano plots and heatmaps (Fig. 3b).

### Cell Types in a High Microbiota Abundance Region (HMAR)

Bacterial location leads to the proliferation of immune cells, such as T cells and B cells, influencing tumor development (Overacre-Delgoffe et al. 2021). SMTdb provides the composition of cell types in a HMAR, which is similar to the microbiota-enriched region (Fig. 3c). Users can identify the co-occurrence of different immune cells and microbiota within a spatial context, which could enable to recognize microbial functions within the TME (Fig. 3c).

### Host–Microbiota Coexpression Module and Clinical Relevance

To understand host–microbiota interactions and elucidate how the microbiota affects the physiological and immune responses of the host, we studied the coexpression modules of the microbiota and host genes in different functional regions (Fig. 3d). SMTdb calculates the microbial gene coexpression modules in each slice. Users can identify the gene modules regulated by microorganisms and the biological functions enriched by them in the spatial slice (Fig. 3d). To investigate the relationship between microbial abundance and prognosis, we performed Cox regression analysis and the log-rank test to identify outcome-associated microbial taxa at the genus level (Fig. 3e).

### Case Study

**Fig. 4.**
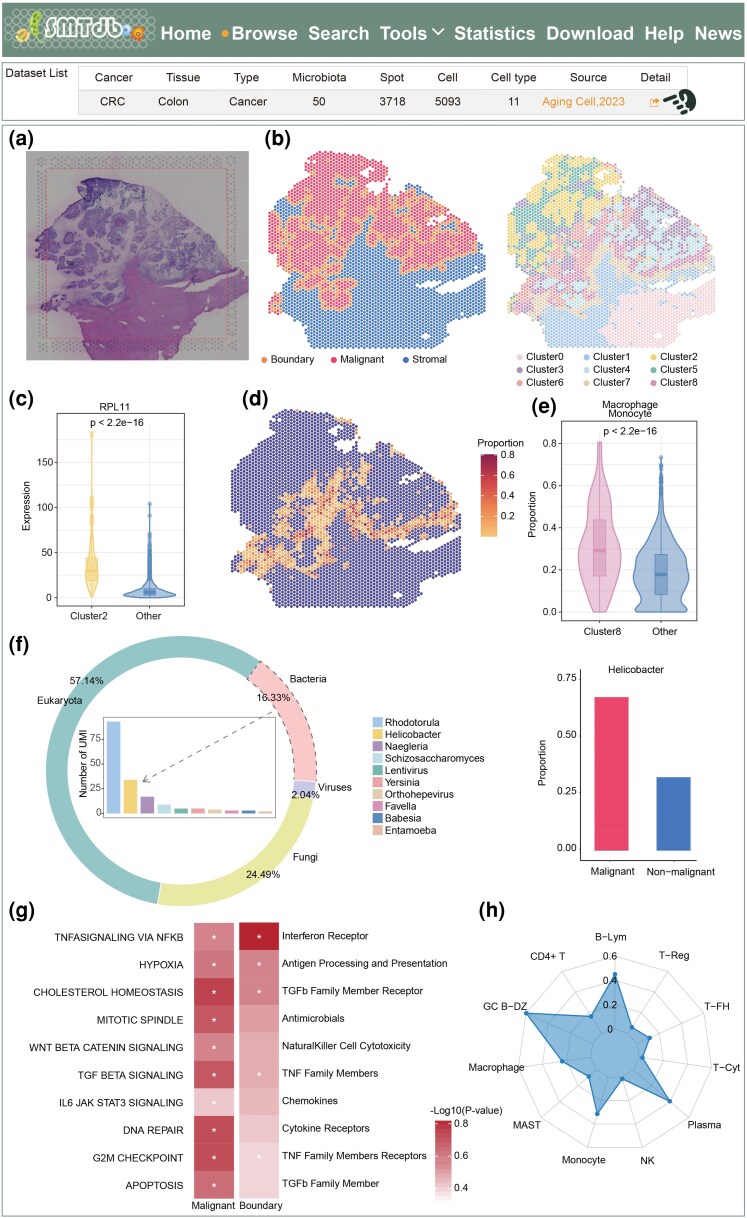
Case study of liver metastasis of CRC patients in SMTdb. a) Hematoxylin-eosin staining image of a tissue slice. b) Spatial neighborhood and transcriptome clusters of a tissue slice. c) Expression of RPL11 between cluster 2 and other clusters (Wilcoxon rank-sum test). d) Distribution of macrophages and monocytes in tissue slices. e) Proportion of macrophages and monocytes in cluster 8 compared to that in other clusters. f) Microbiota composition in tissue slices (left) and *H. pylori* abundance in various spatial neighborhoods (right). g) Cancer hallmarks regulated by the enriched microbiota in malignant spots (left) and immune pathways regulated by the enriched microbiota in boundary spots (right, * represents FDR < 0.05 in the hypergeometric test). h) Immune cells co-occurring with *H. pylori* in the spatial context.

Liver metastasis promotes the main malignant tumor progression events in CRC patients; however, most studies have focused only on the cellular ecosystem of liver tissues. A recent study revealed the remarkable roles of the microbiome in metastatic cancer. Therefore, we used an ST dataset of metastatic liver in CRC to analyze the relevance of the cellular composition and microbiota (Garbarino et al. 2023). Spatial neighborhood and spot transcriptome clusters were generated from tissue slices. Consistent with the senescent metastatic cancer cells in the original study, SMTdb identified the malignant regions and adjacent boundary regions in the same slice (Fig. 4a and b). Parts of spots were annotated as epithelial signature-like metastatic cancer cells (eSMCC) in the original study, which were identical to the spot transcriptome cluster 2 (Fig. 4b, Fig. S3a). Similar to previous results, eSMCC-accumulated RP11 was also significantly overexpressed in cluster 2 (Fig. 4c). Combined with the scRNA-seq reference data, we explored the cellular composition of spatial spots. We found that monocytes dominated malignant boundary regions, which may be recruited by tumor cells (Fig. S3b). Notably, macrophages and monocytes were concentrated mainly at the interface between malignant and stromal regions; this result was highly consistent with the original findings (Fig. 4d, Fig. S3c). Moreover, these cells were enriched in 6, 7, and particularly 8 spot transcriptome clusters, which implied that the transcriptional clusters covered important information on cell-type distribution (Fig. 4e, Fig. S3d).

We also performed metagenomic analysis on another CRC liver metastasis slice. Among all the spots, *Helicobacter pylori* (*H .pylori*) with the second highest abundance was found to be associated with the development of liver disease (Fig. 4f; Sumida et al. 2015; Boziki et al. 2021; Chen et al. 2023b). Additionally, as the most prevalent bacteria in the digestive system (Ralser et al. 2023), the abundance of *H. pylori* in liver tissues might also be due to the spread of CRC cells. As expected, *H. pylori* appeared mainly in the malignant region composed of metastatic cancer cells (Fig. 4f). To investigate the functions of spots with enriched microbiota, we used the specifically upregulated genes in HMAR, which were identified in STMdb, individually for malignant and stromal regions (Fig. S3e). Functional enrichment analysis indicated that cell cycle, DNA repair and TGF-β signaling pathways were upregulated in the malignant cell region, whereas interferon receptor and antigen-related processes were activated in the adjacent boundary regions (Fig. 4g). Co-occurrence analysis using the STMdb data indicated that *H. pylori* was closely associated with monocytes and multiple B lymphocyte subtypes in the spatial context (Fig. 4h), suggesting that their interactions may play a role in the immune response (Peek et al. 2010). A previous study elucidated that *H. pylori* induces the formation of tertiary lymphoid organs, leading to liver inflammation (Shomer et al. 2003). Subsequently, we validated these findings in independent CRC cohorts. In cohort 1, RPL11 was consistently identified as an SVG (Fig. S4a to c). Although RPL11 was not classified as an SVG in cohort 2, it was recognized as a marker gene of spatial cluster 0 (Fig. S4e to f). *H. pylori* also showed consistent enrichment in the malignant regions in both cohorts (Fig. S4g and j), accompanied by the upregulation of cell cycle, DNA repair, and TGF-β signaling pathways. In contrast, the boundary regions also displayed activation of the interferon receptor and antigen-related processes (Fig. S4h and k). Notably, *H. pylori* also exhibited stable spatial colocalization with monocytes and multiple B cell subtypes across both cohorts (Fig. S4i and l). Collectively, these results highlight the reproducibility and robustness of SMTdb.

In summary, based on the annotated ST datasets and analysis pipeline in STMdb, we examined the cellular composition and spatial positioning in CRC liver metastasis slices and assessed the distribution of the microbiota and the co-occurrence between the microbiota and cells, providing a possible interpretation for the liver metastasis of cancer cells and immune-microbial ecosystem in the TME.

## Discussion

This study integrates single-cell omics, ST, and metagenomics data to construct SMTdb, the first comprehensive spatial meta-transcriptome resource for human cancer. This user-friendly platform facilitates the integration of multiomics data, enabling users to determine the expression and spatial distribution of the microbiota and cell types with clarity and precision. SMTdb addresses a critical gap in spatial metatranscriptomic data for cancer research and provides a robust tool to investigate the interactions between microorganisms and the TME. By combining single-cell omics, ST, and metagenomics, SMTdb offers researchers a comprehensive spatial perspective on the role of microorganisms in tumor progression and their interactions with host cells. This facilitates a deeper understanding of microbial niches and functions across the TME and clarifies host–microbiota interactions.

SMTdb contains 203 tissue sections from 20 different cancer types, covering 334,253 spots, 945,149 cells, and 1,218 microbiota. This extensive dataset allows users to compare microbiome differences across cancer types and investigate associations between specific microorganisms and cancer phenotypes. SMTdb contains seven analytical modules, including slice annotation (e.g. spatially co-occurring gene modules, spot deconvolution, and spatially variable genes), microbiota spatial distribution, spatial neighborhood delineation, functional analysis of microbiota-enriched regions, cell-type co-occurrence with bacteria, bacterial–host gene coexpression analysis, and clinical relevance of microbiota. These modules provide insights into the spatial distribution of microorganisms and cell types in tissue sections and reveal their roles and interactions with the TME.

Despite the robust capabilities of SMTdb, it currently includes only FF slices because of limitations related to sequencing technology. Following continuous advancements in sequencing technology, in the future, we will continuously update SMTdb and expand it to other species. In summary, by enhancing the understanding of host–microbiota interactions, SMTdb could serve as a valuable resource that can significantly contribute to the progression of biology and medicine.

## Supplementary Material

msaf263_Supplementary_Data

## Data Availability

All data in the SMTdb can be downloaded from the download page. The source code has been made available on GitHub and can be accessed through the following link: https://github.com/ComputationalEpigeneticsLab/SMTdb.
