# Supplementary material for: SMTdb: A Comprehensive Spatial Meta-Transcriptome Resource in Cancer
Source: Mol Biol Evol. 2025 Oct 15;42(11):msaf263. doi: 10.1093/molbev/msaf263 (PMC12596269; doi:10.1093/molbev/msaf263)
Supplement: msaf263_Supplementary_Data [file msaf263_supplementary_data.zip › Supplementary Materials.pdf]

Supplementary Material: Supplementary Figures and Tables

SMTdb: A comprehensive spatial meta-transcriptome resource in cancer

Weiwei Zhou, Qingyi Yang, Jiyu Guo, Si Li, Minghai Su, Feng Leng, Tingyu Rong, Jingyi Shi, Yueying Gao, Tiantongfei Jiang, Juan Xu, Yongsheng Li

Content

Supplementary Figures S1-S4

Supplementary Tables S1-S5

Supplementary Figures

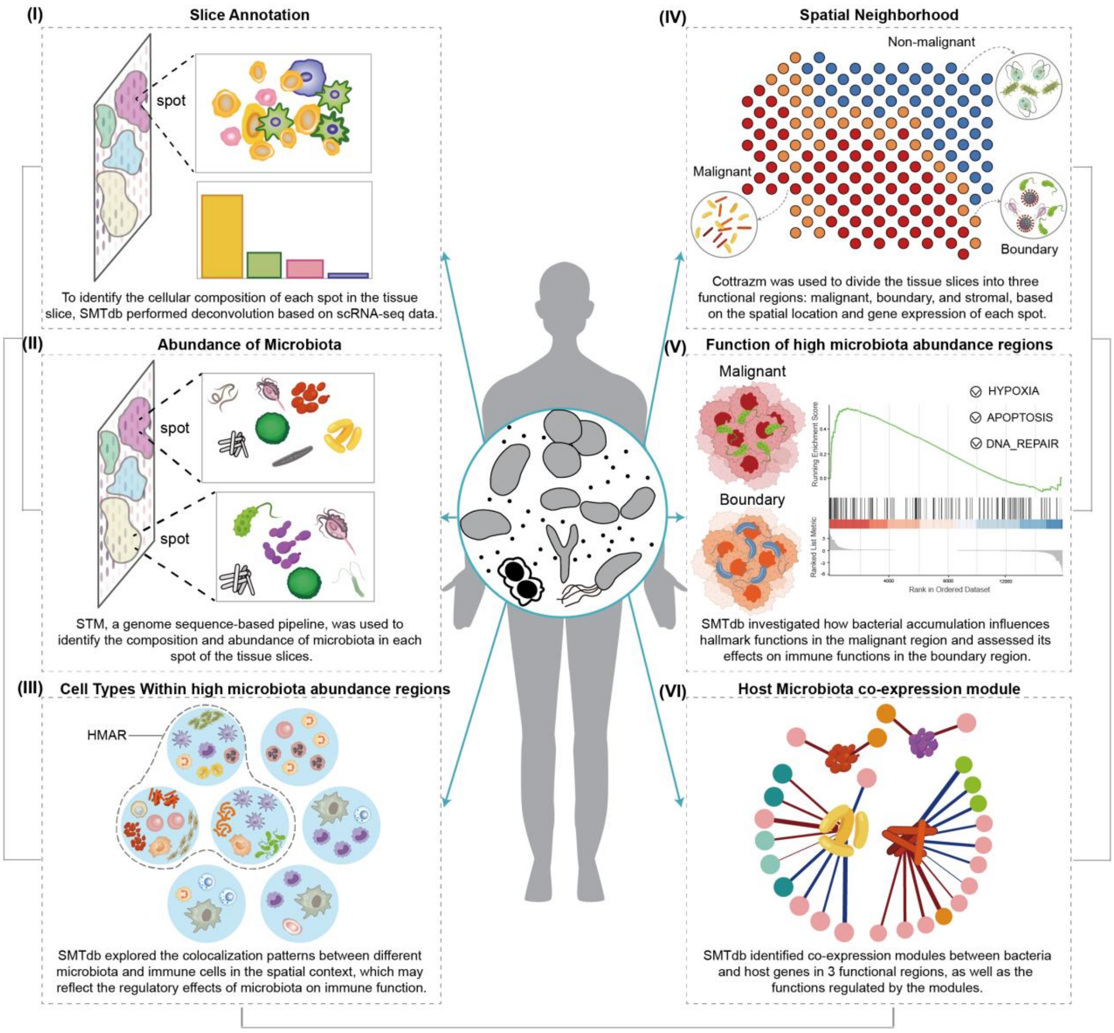

Fig. S1. Functional modules in STMdb.

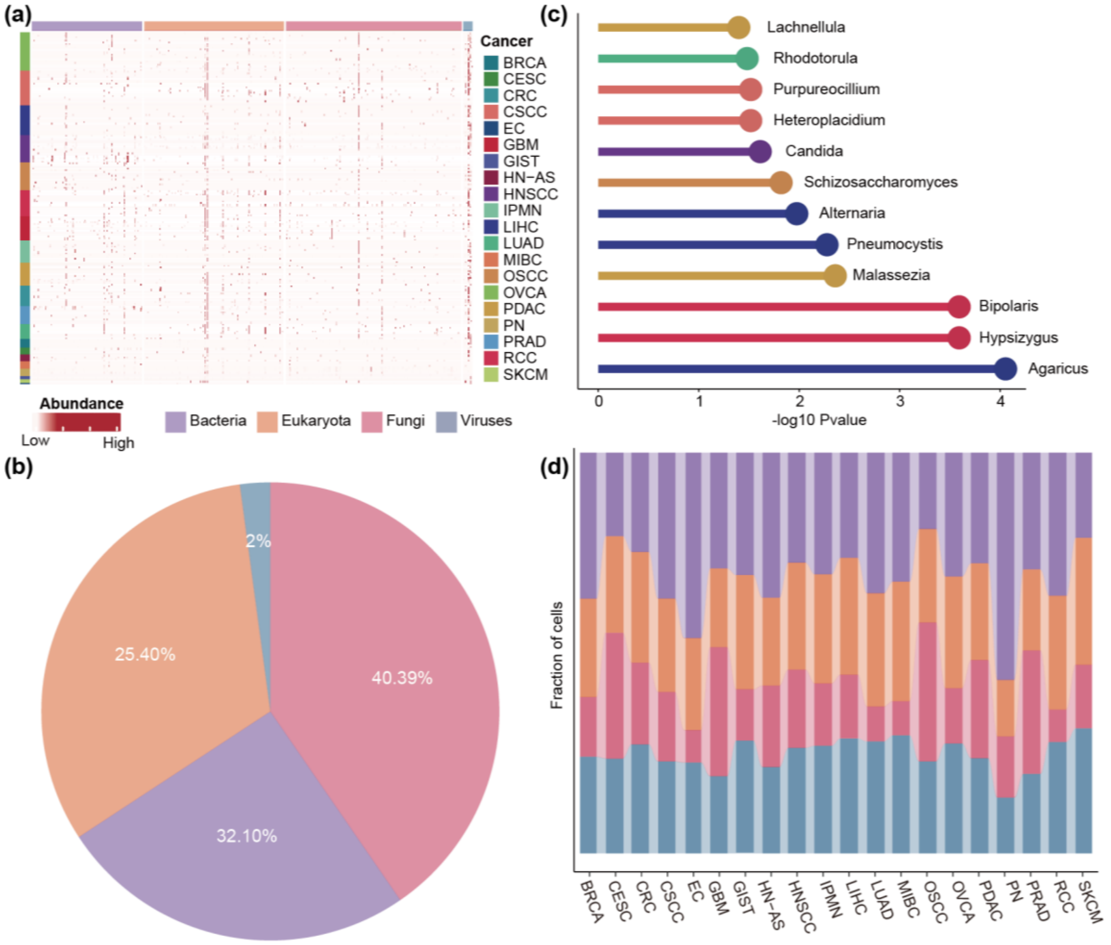

Fig. S2. Spatial meta-transcriptome across different cancer types. (a) Abundance of microbiota across different cancer types. **(b)** The distribution of microbiota in SMTdb. **(c)** Differentially upregulated microbiota identified in tumor tissue slices (Mann-Whitney U test, $p<0.05$). **(d)** The abundance of microbiota in cancer types.

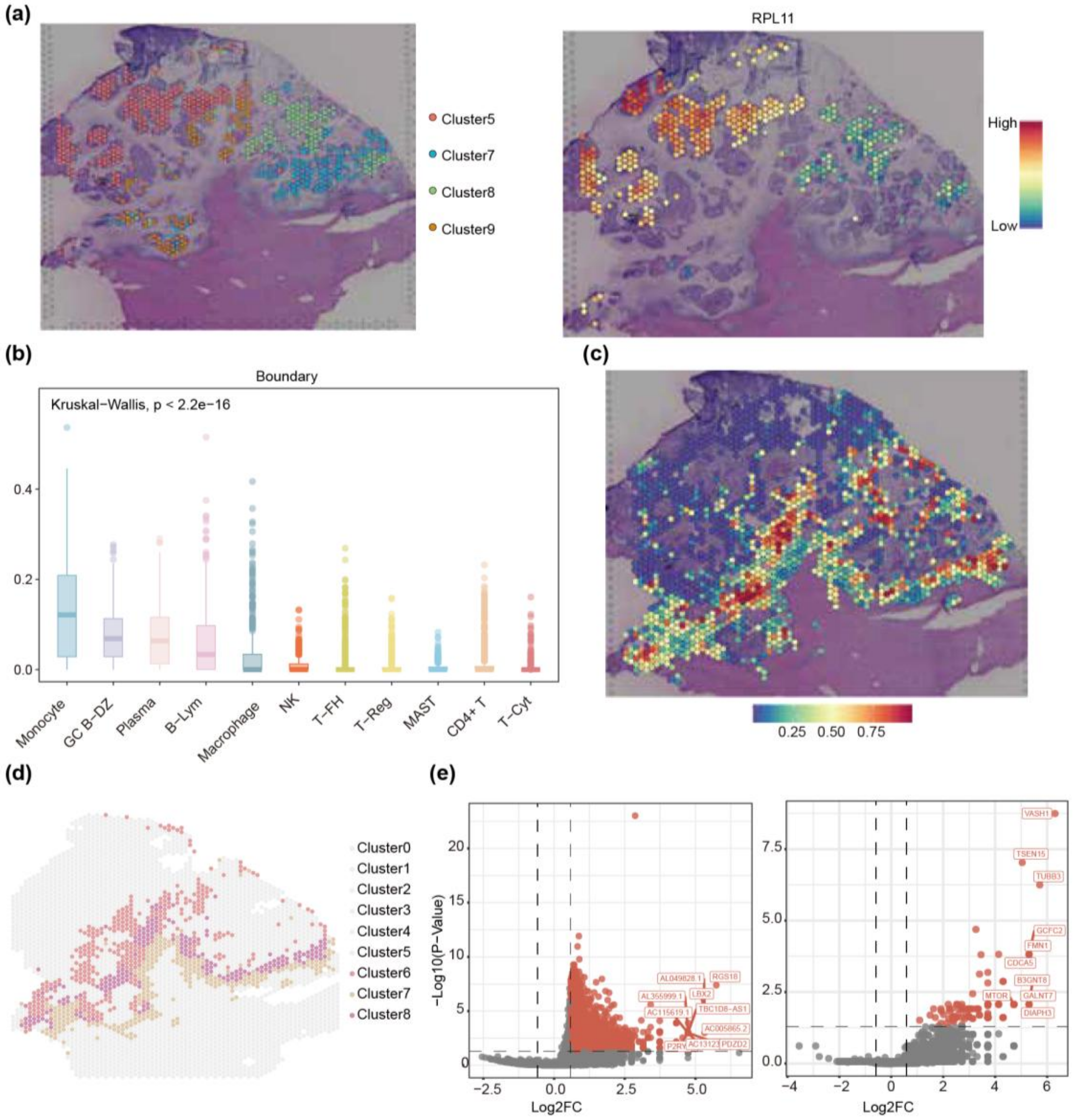

Fig. S3. Case study of colorectal liver metastasis patients in SMTdb. (a) Transcription clusters and expression of RPL11 in original research. **(b)** Composition of immune cells in boundary. **(c)** The distribution of macrophages and monocytes in original research. **(d)** The distribution of cluster 6, 7 and 8 in tissue slice. **(e)** Differentially expressed genes of microbiota enriched region (left: malignant; right: boundary)..

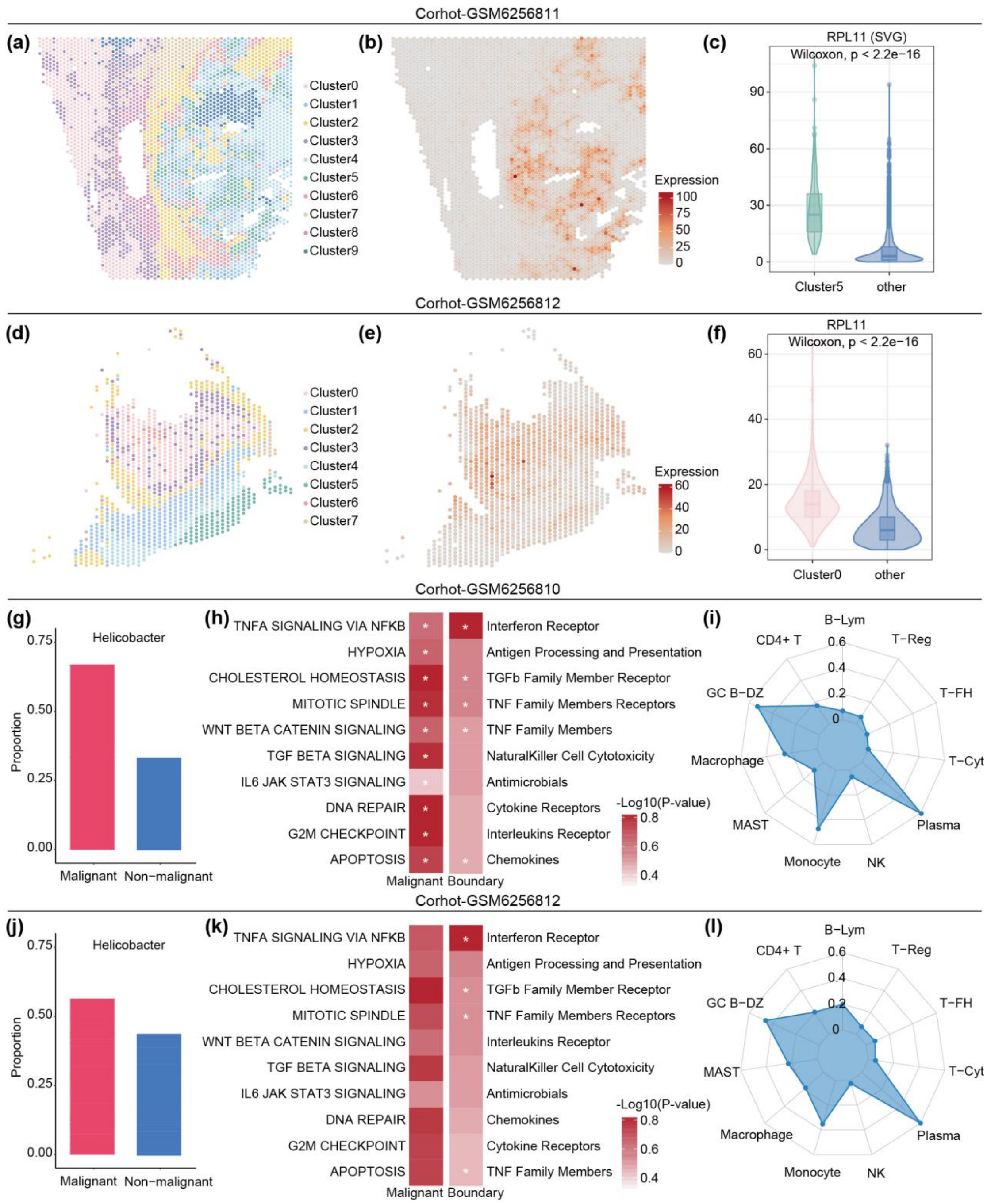

Fig. S4. Validation of the expression of spatial marker gene, distribution of microbiota, functional signatures and gene–microbe co-expression modules in independent cohorts of CRC patients. Transcription clusters and expression of RPL11 in cohort-GSM6256811 **(a-c)** and cohort-GSM6256812 **(d-f)**. The distribution of H.pylori, function of high microbiota abundance neighborhood and immune cells co-occurring with H.pylori in cohort-GSM6256810 **(g-l)** and cohort-GSM6256812 **(j-l)**. **(g,j)** The abundance of H.pylori in various spatial neighborhoods. Red: malignant region, blue: Non-malignant region. **(h,k)** Cancer hallmarks regulated by microbiota enrich in malignant (left) and immune pathways regulated by microbiota enrich in boundary (right, * means FDR<0.05 by hypergeometric test). **(i,l)** Immune cells co-occurring with H.pylori in spatial context.

Supplementary Tables

Supplementary Table S1. The datasets of spatial transcriptome and single cell transcriptome used in SMTdb.

Supplementary Table S2. The lineage map of microbiota in SMTdb.

Supplementary Table S3. The marker gene used in SMTdb.

Supplementary Table S4. The gene set used for the functional enrichment analysis in SMTdb.

Supplementary Table S5. The paired or high quality of scRNA-seq data used in SMTdb.
